# Supplementary material for: Comparative Transcriptome Analysis Reveals the Effects of a High-Protein Diet on Silkworm Midgut
Source: Insects. 2025 Mar 24;16(4):337. doi: 10.3390/insects16040337 (PMC12027703; doi:10.3390/insects16040337)
Supplement: Supplementary file 1 [file insects-16-00337-s001.zip › Table S1 qPCR primers.pdf]

Table S1. qPCR primers

Gene name	Sequence of sense primer (5'→3')	Sequence of anti-sense primer (5'→3')
COX2	ATCGGACATCAATGATACTGAA	CGACCTGGATTAGCATCAACT
Vha100-2	TGCCAACATAACGGCACGAT	GTTGGCACAAAGCCATCTCC
ATPsynCF6	AGAAGCAGTATGGAGGGGGT	TGGGGTCCAGCTTAGGTTCT
ND5	GTCTTTCGACCGTTGCGTTG	AGGTCTAAGGTGAGGGGAGG
ND-13B	CGCAAATCCGCATCACACTT	AACCGCTGCTCTTTCACGTA
BMCYCvb	CGCCACTTGCTATCGTGAGA	CGGGTTCCTGCTGACTTCTT
