# Supplementary material for: Comparative Transcriptome Analysis Reveals the Effects of a High-Protein Diet on Silkworm Midgut
Source: Insects. 2025 Mar 24;16(4):337. doi: 10.3390/insects16040337 (PMC12027703; doi:10.3390/insects16040337)
Supplement: Supplementary file 1 [file insects-16-00337-s001.zip › Table S2 Investigation on the weight of 20 female silkworms(g).pdf]

Table S2. Investigation on the weight of 20 female silkworms(g)

Day	Control			HPD6%			HPD12%		
5L1D	20.42	20.36	21.32	20.48	20.53	21.99	20.12	20.36	20.95
5L2D	28.42	27.3	28.53	31.12	29.6	30.08	29.07	29.58	29.73
5L3D	43.17	43.64	42.82	46.34	47	46.96	43.32	43.36	42.91
5L4D	64.67	63.08	64.98	66.7	67.23	66.89	64.16	64.76	65.02
5L5D	76.58	75.49	76.81	81.51	81.92	81.83	76.03	76.8	76.56
5L6D	90.29	91.01	89.88	95.77	95.8	96.23	88.05	88.08	88.86
