# Supplementary material for: Comparative Transcriptome Analysis Reveals the Effects of a High-Protein Diet on Silkworm Midgut
Source: Insects. 2025 Mar 24;16(4):337. doi: 10.3390/insects16040337 (PMC12027703; doi:10.3390/insects16040337)
Supplement: Supplementary file 1 [file insects-16-00337-s001.zip › Table S3 Quality control.pdf]

Table S3: Quality control

Sample	D10/Control	D102/Contro12	D103/Contro13	B10/HPD1	B102/HPD2	B103/HPD3
raw reads	50376714	52403276	55850698	48040298	49054940	50762138
raw bases	7.56G	7.86G	8.38G	7.21G	7.36G	7.61G
clean reads	47415706	49425026	52128876	45997240	47017672	48576008
clean bases	7.11G	7.41G	7.82G	6.9G	7.05G	7.29G
clean reads ratio (%)	94.12	94.32	93.34	95.75	95.85	95.69
Q20(%)	99.14	99.13	99.14	99.03	99.07	99.15
Q30(%)	97.45	97.38	97.46	97.22	97.25	97.43
GC content (%)	49.01	47.34	47.21	45.19	43.71	43.94
