# Supplementary material for: Comparative Transcriptome Analysis Reveals the Effects of a High-Protein Diet on Silkworm Midgut
Source: Insects. 2025 Mar 24;16(4):337. doi: 10.3390/insects16040337 (PMC12027703; doi:10.3390/insects16040337)
Supplement: Supplementary file 1 [file insects-16-00337-s001.zip › Table S4 List of gene set enrichment analysis statistics.pdf]

Table S4. List of gene set enrichment analysis statistics

Gene set name	Description	SIZE	ES	NES	NOM p-val	FDR q-val	FWER p-val	RANK AT MAX	LEADING EDGE
BMOR03030	DNA replication	35	0.773905	1.493534	0	0.142634	0.426	2420	tags=60%, list=13%, signal=69%
BMOR03010	Ribosome	123	0.756272	1.35988	0	0.191252	0.904	3359	tags=73%, list=19%, signal=89%
BMOR00190	Oxidative phosphorylation	155	0.713625	1.380527	0	0.191259	0.904	2991	tags=58%, list=17%, signal=69%
BMOR03410	Base excision repair	30	0.655438	1.36528	0.137177	0.194288	0.904	2666	tags=57%, list=15%, signal=66%
BMOR03430	Mismatch repair	20	0.758436	1.388953	0	0.210859	0.904	3771	tags=70%, list=21%, signal=89%
BMOR03460	Fanconi anemia pathway	31	0.514433	1.329624	0	0.219374	0.904	3771	tags=52%, list=21%, signal=65%
BMOR03040	Spliceosome	119	0.576031	1.451242	0	0.219869	0.628	3711	tags=54%, list=21%, signal=67%
BMOR00970	Aminoacyl-tRNA biosynthesis	36	0.719002	1.397204	0	0.222494	0.795	2766	tags=58%, list=15%, signal=69%
BMOR03420	Nucleotide excision repair	52	0.59361	1.414989	0	0.243291	0.795	3725	tags=50%, list=21%, signal=63%
BMOR03008	Ribosome biogenesis in eukaryotes	68	0.719674	1.494838	0	0.245267	0.386	1864	tags=54%, list=10%, signal=60%

GS：Name of gene set, name of kegg pathway.

SIZE：The kegg pathway contains the number of genes in the expression dataset text (the value after conditional screening).

ES：Enrichment score.

NES：Normalized es value after correction. Since the number of gene sets in the gene database files input by different users may be different, the number and size of gene sets are considered in the standardization of enrichment score. Its absolute value greater than 1 is an enrichment standard.

NOM p-val：P-value is the statistical analysis of enrichment score es, which is used to characterize the reliability of enrichment results.

FDR q-val：That is, Q-value is the p-value after multiple hypothesis test correction, that is, the probability estimate of the possible false positive results of NES, so the more obvious the FDR is, the more significant the enrichment is.

RANK AT MAX：When the ES value * *, the position of the corresponding gene in the sorted gene list.

LEADING EDGE：There are three statistical values here, with tags=59% indicating the percentage of core genes in the total number of genes in the gene set; List=21% indicates the percentage of core genes in all genes.
