# Supplementary material for: Comparative Transcriptome Analysis Reveals the Effects of a High-Protein Diet on Silkworm Midgut
Source: Insects. 2025 Mar 24;16(4):337. doi: 10.3390/insects16040337 (PMC12027703; doi:10.3390/insects16040337)
Supplement: Supplementary file 1 [file insects-16-00337-s001.zip › Table S5 Gene expression in oxidative phosphorylation pathway.pdf]

Table S5：Gene expression in oxidative phosphorylation pathway

Up-regulated and down-regulated genes	Gene Numbers	Gene Name	Gene ID	B10/HPD1	B102/HPD2	B103/HPD3	D10/Control1	D102/Control2	D103/Control3	Log2FoldChange	Pvalue	Remark
Up-regulated genes in complex I	18	ND1	809263	3075.00906	5637.901509	1976.119311	1018.480788	1075.442882	1439.497233	1.596724343	4.19E-06	
			novel.875	5434.852484	7876.786531	2511.44575	811.5810209	1857.821037	1420.721183	1.951149517	7.30E-07	New gene
		ND2	119631129	104.6336086	152.8316903	42.82611511	16.01804646	31.39979218	53.1988108	1.565949841	0.004444737	
		ND4	809259	115.8080716	187.5661653	85.65223021	36.04060455	48.84412117	44.8538993	1.575301518	7.36E-05	
			119630859	203.1720555	97.25653018	30.59008222	28.03158131	30.52757573	31.29341812	1.876400025	0.001222984	bmor: 119630859: No data in KEGG Mapper
		ND5	809258	154.4107622	257.0351155	107.8300398	50.72381381	80.24391334	106.3976216	1.122315364	0.006121963	
		NDUFs4	101739007	1523.790416	1464.802434	1807.109107	1182.665764	1148.709064	1157.85647	0.459311299	0.014672462	
		NDUFs6	101741774	880.7508605	796.9080993	849.6395336	549.9529286	600.9571336	656.1186665	0.483279298	0.012703977	
		Ndufa4	119630916	3628.652911	3130.07241	3297.610863	2392.028272	2221.535297	2562.930944	0.486823631	0.006786514	bmor: 119630916: No data in KEGG Mapper
		Ndufa2	693085	806.5930603	883.2480802	954.4105652	543.2787426	564.3240427	622.7390206	0.61183526	0.00182002	
		Ndufa5	100101189	719.2290764	689.7274334	950.586805	439.1614406	464.8913675	476.7030693	0.773489783	0.00046108	
		Ndufab1	733017	3169.484066	2845.249714	3470.444828	2293.250319	2432.611677	2471.136917	0.398294051	0.027653733	
		Nd-13B	692866	2931.772761	2669.592512	2937.412645	2313.272877	2083.725098	2321.971624	0.34604897	0.047851042	
		Ndufb3	101737774	1765.565162	2054.296097	1940.175965	1564.429205	1315.302406	1551.110425	0.378993814	0.044605275	
		Ndufb6	778510	1440.489873	1562.058964	1713.809356	1150.629671	1101.609376	1169.330724	0.463449713	0.01123896	
		Ndufb7	101741007	1322.650081	1371.515558	1423.203575	1113.254229	976.0102069	1055.631305	0.389430999	0.03070362	
		Ndufb9	101739528	2176.988575	2226.976058	2385.261661	1412.257763	1219.358596	1313.280447	0.783915766	1.18E-05	
		Ndufc2	101745713	716.1814956	765.150865	829.7559802	557.9619519	629.7402765	586.2300327	0.381254926	0.049426383	
Down-regulated genes in complex I	1	Ndufa6	101738818	0	0	0	10.67869764	3.488865798	0	-4.79419028	0.044446838	
Up-regulated genes in complex II	2	SDHD	101737567	3384.846444	3265.040656	4456.974979	2562.887434	2733.526352	2658.897426	0.481582556	0.017240626	
		SDHB	101746165	3166.436485	3596.506789	5448.093643	2771.122038	2961.174846	2876.908239	0.504400105	0.038691788	

**Up-regulated genes in complex III**	**10**	QCR6	101736448	2261.304978	2454.238767	2762.284424	1571.103391	1575.222908	1664.809844	0.636422725	0.000496844	
		QCR8	101745824	1530.901438	1580.914822	1544.799152	1138.616136	1104.226025	1288.245713	0.399179656	0.0270182	
		Cytb	119630863	1208.87373	3195.571706	2415.086991	120.1353485	296.5535928	458.9701324	2.95921484	1.18E-11	bmor: 119630863: No data in KEGG Mapper
			119630861	3493.543494	9060.735924	2319.492984	668.7534399	580.0239388	1103.614546	2.660493377	6.53E-10	bmor: 119630861: No data in KEGG Mapper
			119630862	2482.762518	5780.809064	1974.589807	521.9213473	634.9735751	1026.424114	2.22888722	1.29E-08	bmor: 119630862: No data in KEGG Mapper
			119631130	165.5852252	245.1261526	50.47363566	40.04511616	38.37752377	52.15569686	1.817569969	0.000353214	bmor: 119631130: No data in KEGG Mapper
			809262	669.4519228	1364.568663	514.6781333	154.8411158	206.7152985	244.0886613	2.070959519	1.25E-08	
		Cyt1	100127128	6486.267871	6916.130192	8923.891735	5586.293705	5433.90848	5110.215179	0.469096806	0.01932467	
		QCR7	751613	3579.891618	3302.752372	3589.746148	2468.113993	2596.58837	2546.241121	0.460372808	0.006217908	
		QCR10	100484985	1700.550104	1912.380956	1809.403363	1293.457252	1250.758388	1293.461282	0.498819818	0.004358015	
**Up-regulated genes in complex IV**	**13**	COX1	119630858	45287.05117	78079.13029	33222.35879	3991.163244	5751.395267	7050.407102	3.220941526	1.06E-22	bmor: 119630858: No data in KEGG Mapper
			110386648	7608.793478	12030.03733	7158.079239	1859.428227	766.678259	1322.668472	2.76344596	6.26E-17	bmor: 110386648: No data in KEGG Mapper
			119630857	66888.30411	153415.2295	76986.82467	9589.470484	10926.25546	20236.41038	2.866901828	2.53E-15	bmor: 119630857: No data in KEGG Mapper
			119631133	6449.696901	7616.774174	4845.469023	1034.498834	2484.072448	1636.645768	1.874399453	3.67E-09	bmor: 119631133: No data in KEGG Mapper
			809266	2673.74425	9030.963516	2607.039757	612.6902773	776.2726399	1015.992975	2.57275734	1.71E-09	
		COX2	809267	19.30134527	45.65102437	45.88512333	8.009023232	12.21103029	12.51736725	1.749612017	0.003963733	
		COX4	780848	14745.21193	15181.94284	15345.51475	11587.72178	11597.86213	12379.67621	0.348172723	0.031749453	
		COX5B	100134926	8409.291377	8131.836819	10074.07883	6134.911796	6401.196522	6354.650106	0.494610368	0.006265164	
		COX6C	101746562	4668.893835	4949.16649	5100.89621	3897.72464	3815.946966	3705.140705	0.366354362	0.027457391	
		COX7C	101743428	4813.145994	4907.48512	3949.944367	3103.496503	2451.800439	3393.249638	0.611455512	0.003827465	
		COX8	101745824	1530.901438	1580.914822	1544.799152	1138.616136	1104.226025	1288.245713	0.399179656	0.0270182	bmor: 101745824: No data in KEGG Mapper
		COX17	101743531	1718.835589	1318.917639	2319.492984	978.4356716	1207.147566	1197.4948	0.663094824	0.009008022	bmor: 101743531: No data in KEGG Mapper
		CYC	101738358	8098.438132	7832.12792	10293.56267	6240.363935	6920.165309	6625.85973	0.40640704	0.03641113	
**Up-regulated genes in complex V**	**15**	ATP6	809269	539.4218073	1569.998273	351.7859455	197.5559064	169.2099912	267.0371679	1.957006647	1.87E-05	
		ATP8	809268	12.19032333	29.7724072	9.177024666	8.009023232	1.744432899	5.215569686	1.809597972	0.044852132	

		ATP-c	101738009	30987.8019	36607.15948	42849.82242	30344.85419	26350.53115	27271.17078	0.395477711	0.047216226	
		ATP-delta	100101165	5157.522629	5016.650613	5362.441413	3897.72464	3881.3632	3903.332353	0.411377639	0.011859309	
		ATP-d	100101181	8001.931405	8738.201512	9542.576148	6672.85119	6537.262288	7171.408319	0.366911294	0.036731939	
		ATP-e	778517	4226.994614	3912.094306	3866.586392	2687.027294	2517.216673	2986.435202	0.551681285	0.001920106	
		ATP-g	732934	3134.944816	3103.277243	3418.441688	2310.603203	2126.463704	2545.198007	0.468035808	0.009416815	
		ATPsynCF6	778519	5031.555954	4556.170715	4839.351007	3273.020828	2783.24269	3493.388576	0.595442024	0.001284438	
		ATP-f	101744632	6315.603345	5880.050421	5913.827645	4187.384313	4357.593381	4835.876213	0.436483887	0.011332029	
		VATPc	692859	15508.123	18675.23862	22591.54047	14127.91698	15062.30586	13699.21534	0.404657055	0.048892659	
		ATP-G	692985	25320.31742	27233.81328	30339.24354	20891.5371	20222.33838	18595.59216	0.473328202	0.007410257	
		Vha100-2	101742516	18808.65304	24261.53462	28451.83547	22209.02142	12781.45985	13230.85718	0.568813878	0.038434166	
		ATP-S1	101741915	28884.97113	36106.98303	37164.65564	26256.24783	25402.43187	21767.70164	0.476434738	0.01505611	
		Nurf-38 PE	101741235	1877.309793	2007.652659	2958.060951	1679.225204	1262.969419	1685.672123	0.565019599	0.023420731	
		ATP-epsilon	101740954	2440.096386	2273.619496	2506.857238	1705.921948	1611.855998	1878.648201	0.474763532	0.007953469	
