# Supplementary material for: Comparative Transcriptome Analysis Reveals the Effects of a High-Protein Diet on Silkworm Midgut
Source: Insects. 2025 Mar 24;16(4):337. doi: 10.3390/insects16040337 (PMC12027703; doi:10.3390/insects16040337)
Supplement: Supplementary file 1 [file insects-16-00337-s001.zip › Table S6 Gene expression in nuclear transcription pathway.pdf]

Table S6: Gene expression in nuclear transcription.

KEGG Pathway	Up-regulated and down-regulated genes	Gene Number	Gene Name	Gene ID	B10/HPD1	B102/HPD2	B103/HPD3	D10/Control1	D102/Control2	D103/Control3	Log2FoldChange	Pvalue
Ribosome	Up-regulated genes in robosomal small subunit	19	mRpS17	101737237	372.8207218	346.3523371	572.7992895	162.8501391	227.6484933	185.6742808	1.163420769	5.07E-05
			mRpS10	732896	188.9500116	214.3613318	248.544418	80.09023232	132.5769003	91.79402648	1.091810481	0.000304
			mRpS5	732873	1148.937974	1046.003906	1369.670931	696.7850212	742.2561984	799.025276	0.671591168	0.001014
			mRpS7	101739965	586.1513801	588.5012489	845.8157734	307.0125572	408.1972983	437.0647397	0.809362741	0.001646
			mRpS2	101739031	224.5051213	198.4827146	319.6663592	138.8230694	136.0657661	134.5616979	0.861731639	0.002281
			mRpS15	101746056	410.4075521	347.3447506	659.2162718	193.5513948	314.8701382	239.9162056	0.919486554	0.003513
			mRpS6	101740286	262.0919516	332.458547	342.6089209	160.1804646	212.8208136	198.1916481	0.711708338	0.004862
			mRpS9	101745649	420.5661549	447.5785215	767.8110637	327.0351153	324.4645192	286.8563328	0.80358607	0.005023
			mRpS12	733035	549.5804101	598.4253847	894.7599049	369.7499059	491.057861	418.2886888	0.675049019	0.010477
			mRpS11	101741005	266.1553927	327.4964792	312.7835907	208.234604	200.6097834	196.1054202	0.584471525	0.011112
			RpSA	692424	9257.534708	9784.205418	9866.83102	7174.749979	5652.834808	7716.956908	0.492864723	0.011761
			mRpS14	100101170	257.0126502	245.1261526	256.1919386	181.5378599	167.4655583	165.855116	0.560252713	0.015037
			RpS24	100101150	5440.947646	4943.212008	5298.966992	3166.233851	3386.816473	4563.623476	0.496381906	0.018442
			RpS20	778514	6793.057675	6564.815787	6957.714201	5119.100683	4170.939061	5576.487109	0.450670289	0.018679
			mRpS21	732874	345.3924943	301.6937263	462.6749936	208.234604	280.8536967	267.0371679	0.552520702	0.038081
			RpS21	692689	2903.328673	2679.516648	2336.317529	1705.921948	1479.279098	2417.938107	0.49905173	0.038163
			mRpS18C	100101195	145.2680197	122.0668695	211.8363194	72.08120909	99.43267523	122.0443307	0.705354644	0.04011
			mRpS18	101736005	313.9008257	388.0337071	465.7340018	237.6010226	294.8091599	286.8563328	0.510646203	0.041359
			RpS12	732856	6582.774598	6541.990275	5594.926038	4434.329196	4334.915753	5546.236804	0.386893688	0.049264
	Up-regulated genes in robosomal large subunit	31	mRpL12	733111	671.4836434	622.2433104	730.338213	343.0531618	385.5196706	397.4264101	0.84529036	3.44E-05
			mRpL35	101742782	371.8048615	349.3295778	494.7945799	200.2255808	201.4819998	218.0108129	0.973631115	7.04E-05
			mRpL27	101741251	230.600283	220.3158133	249.3091701	120.1353485	135.1935497	138.7341537	0.827846723	0.000759

				mRpL1	101736359	358.5986779	309.6330348	533.7969347	221.5829761	221.5429781	197.1485341	0.910622756	0.00101
				mRpL22	101745675	201.1403349	289.7847634	280.6640044	157.5107902	134.3213332	126.2167864	0.887817806	0.001092
				mRpL13	101737803	469.3274482	373.1475035	527.6789183	237.6010226	248.5816881	304.5892697	0.793119649	0.00123
				mRpL21	101736166	202.1561952	230.239949	232.4846249	140.1579066	121.2380865	123.0874446	0.793703586	0.001523
				mRpL32	778516	400.2489493	349.3295778	270.7222276	180.2030227	199.7375669	210.7090153	0.785661052	0.00197
				mRpL10	101739700	495.7398154	381.0868121	661.510528	230.9268365	347.1421469	252.4335728	0.887774542	0.002096
				mRpL19	101736455	376.8841629	334.4433742	491.7355717	182.8726971	268.6426664	230.5281801	0.816541955	0.002298
				RpL20	692841	236.6954446	303.6785534	300.5475578	161.5153019	186.6543202	164.8120021	0.711905686	0.003875
				mRpL34	101743322	340.3131929	262.9895969	390.0235483	158.8456274	226.7762768	197.1485341	0.766758336	0.004552
				mRpL11	101736605	214.3465185	191.5358196	344.138425	72.08120909	167.4655583	125.1736725	1.034870921	0.005277
				mRpL23	101744664	336.2497518	432.6923179	439.7324319	220.2481389	293.9369434	244.0886613	0.670523395	0.006053
				mRpL4	101746143	475.4226098	437.6543858	601.8598677	311.0170689	327.0811685	351.5293969	0.614898387	0.007154
				RpL34	100101149	4071.567992	3946.828781	3512.506191	2473.453342	2299.162561	3138.729837	0.543446026	0.00774
				mRpL9	101746284	459.1688454	379.101985	603.3893718	273.6416271	334.0589001	297.2874721	0.671537648	0.008547
				mRpL18	101745044	121.9032333	112.1427338	216.4248317	78.75539512	91.58272718	66.75929199	0.927888286	0.010051
				RpL36A	778454	3765.794048	3830.716393	2876.997233	2171.780133	2082.852881	2877.951353	0.55406678	0.013725
				mRpL17	101744946	276.3139955	207.4144368	263.8394591	133.4837205	179.6765886	164.8120021	0.641737013	0.015194
				RpL27	692703	6113.44715	5487.054646	4807.996173	3454.558688	3394.666421	4642.900135	0.513682751	0.016831
				mRpL24	101743958	604.4368651	558.7288417	788.4593692	468.5278591	450.9359043	433.9353979	0.52944483	0.018347
				mRpL30	101739135	423.6137357	388.0337071	561.3280087	246.944883	329.6978179	334.8395739	0.589967838	0.021028
				RpL35	692706	5677.643091	5424.532591	4657.340018	3407.839385	3483.632499	4499.993525	0.468119923	0.022359
				RpL14	692669	12645.42873	12007.21182	11029.25414	8596.351603	8974.235048	10149.49861	0.36419331	0.040749
				RpL35A	692707	6428.363836	6909.183297	6950.06668	4934.893148	5254.231891	5726.695516	0.350083386	0.0423
				mRpL15	101742656	600.373424	577.5846996	794.5773856	428.4827429	515.4799216	498.608462	0.451231391	0.047999
				RLP24	101746755	536.3742265	492.2371323	643.9212307	357.736371	401.2195667	465.228816	0.450049148	0.048188

			RpP0	692657	10623.86678	11529.86089	11192.91108	7764.748024	6845.154695	7815.009618	0.57252244	0.000822
			RpP1	778451	8358.498363	8013.739604	6502.686728	5024.327241	5423.441882	6410.978258	0.44012088	0.029713
			mRpL2	733060	738.5304217	695.6819148	967.4113502	596.6722308	593.979402	597.7042861	0.426279198	0.049516
**Ribosome biogenesis in eukaryotes**	**Up-regulated genes in Ribosome biogenesis in eukaryotes**	25	GNL3	101736688	259.0443707	202.4523689	326.5491277	130.8140461	144.7879306	105.3545077	1.049434288	0.000326
			DKC1	101744752	136.1252772	183.596511	288.3115249	92.10376717	89.83829429	77.19043136	1.234193442	0.000355
			RRP7A	101745950	100.5701675	105.1958388	72.65144527	26.69674411	42.73860602	46.94012718	1.244724818	0.001303
			RIO1	101739095	189.9658719	193.5206468	190.4232618	90.76892997	118.6214371	112.6563052	0.828292014	0.001747
			RBP28	101735989	155.4266224	144.8923817	184.3052454	58.73283704	101.1771081	85.53534286	0.973604585	0.002131
			NHP2	732976	69.07849887	80.38549943	106.3005357	42.71479057	40.12195667	39.63832962	1.067922345	0.003768
			NXF1	101739030	639.9919748	528.9564345	507.7953648	317.6912549	401.2195667	354.6587387	0.640697768	0.004095
			FBRL	101742289	698.9118709	608.3495204	1061.475853	468.5278591	531.1798177	369.2623338	0.79153742	0.00448
			REXO5	101738143	144.2521594	103.2110116	102.4767754	49.3889766	70.6495324	60.50060836	0.945618015	0.005128
			BMS1	101745253	179.8072691	193.5206468	289.841029	150.8366042	108.1548397	107.4407355	0.863087075	0.005371
			RpL7Ae	692972	105.6494689	114.1275609	84.88747816	58.73283704	58.43850211	43.81078537	0.919003571	0.006893
			MPP10	101745671	93.45914552	116.1123881	139.1848741	69.41153468	51.46077051	66.75929199	0.902691158	0.006944
			IMP4	101736210	61.96747692	40.6889565	100.1825193	21.35739529	20.06097834	41.72455749	1.289940223	0.01121
			RPP25	101745517	65.01505776	47.63585151	72.65144527	38.71027896	22.67762768	30.25030418	1.031789169	0.016323
			NAT10	101744824	153.3949019	146.8772088	259.2509468	116.1308369	104.6659739	108.4838495	0.769651226	0.016335
			TBL3	101736004	110.7287702	131.9910052	168.2454522	66.74186027	77.62726399	97.00959617	0.76731384	0.017794
			NOG2	101746403	177.7755486	195.5054739	302.841814	130.8140461	140.4268483	151.2515209	0.68018643	0.022758
			FCF1	101745565	16.25376444	23.81792576	37.47285072	9.343860438	6.105515146	12.51736725	1.487664345	0.026859
			NOB1	101737534	157.458343	192.5282332	300.5475578	85.42958115	151.7656622	143.9497233	0.768414945	0.028766
			WD	101736458	178.7914088	203.4447825	281.4287564	162.8501391	121.2380865	146.0359512	0.631668268	0.030059
			RCL1	101737764	58.91989609	55.5751601	65.00392472	49.3889766	22.67762768	19.81916481	0.987808215	0.035435
			NOP56	101737128	455.1054043	284.8226955	469.5577621	282.9854875	308.7646231	207.5796735	0.597943372	0.037905

			POP1	101745003	44.69785221	25.8027529	69.59243705	20.02255808	20.06097834	26.07784843	1.087661078	0.038684
			RAN	692970	2461.429452	2146.590559	2347.78881	1629.836228	1999.120102	1665.852958	0.393182307	0.038707
			UTP6	101739847	67.04677831	66.49170941	90.24074255	25.3619069	46.22747182	52.15569686	0.845932469	0.039588
**Base excision repair**<br>**Mismatch repair**<br>**DNA replication**	**Up-regulated genes in Base excision repair, Mismatch repair, and DNA replication**	**13**	Pcna	692356	121.9032333	172.6799617	271.4869797	66.74186027	55.82185276	51.11258293	1.711478533	3.81E-06
			FEN1	101739719	164.5693649	101.2261845	86.41698227	46.71930219	51.46077051	42.76767143	1.316531841	0.000481
			RRP1	101741812	215.3623788	192.5282332	380.8465236	138.8230694	121.2380865	115.785647	1.073760276	0.001049
			DPOD1	101735729	78.22124136	111.1503202	119.3013207	60.06767424	43.61082247	56.32815261	0.956746107	0.006721
			GATC	101741322	70.09435914	83.36274015	74.18094938	24.0270697	46.22747182	42.76767143	0.995345153	0.012348
			DNLI1	692827	266.1553927	206.4220232	210.3068153	108.1218136	151.7656622	162.7257742	0.686857148	0.014389
			NTH	732919	96.50672636	82.37032658	189.6585098	53.39348822	75.01061465	60.50060836	0.963847332	0.017359
			RFC5	101739080	268.1871132	257.0351155	469.5577621	214.9087901	168.3377747	220.0970408	0.725168741	0.018999
			DPOE1	101739562	48.76129332	53.59033295	52.76789183	10.67869764	33.14422508	23.99162056	1.169459544	0.019283
			SSBP	101746319	42.66613165	50.61309223	104.0062795	30.70125572	38.37752377	15.64670906	1.221765839	0.019734
			MCM5	692838	101.5860277	136.9530731	138.420122	54.72832542	105.5381904	59.45749442	0.770121531	0.03497
			Mcm7	692463	93.45914552	131.9910052	153.7151631	108.1218136	61.05515146	32.33653206	0.922921642	0.037045
			PMS2	101742003	108.6970497	164.7406532	209.5420632	86.76441835	124.7269523	86.57845679	0.694646393	0.04457
	**Down-regulated genes in Base excision repair**	1	PARG	101742248	41.65027137	32.74964792	39.76710688	72.08120909	65.4162337	77.19043136	-0.908803883	0.013333
