# Supplementary figures and images for: Comparative Transcriptome Analysis Reveals the Effects of a High-Protein Diet on Silkworm Midgut
Source: Insects. 2025 Mar 24;16(4):337. doi: 10.3390/insects16040337 (PMC12027703; doi:10.3390/insects16040337)

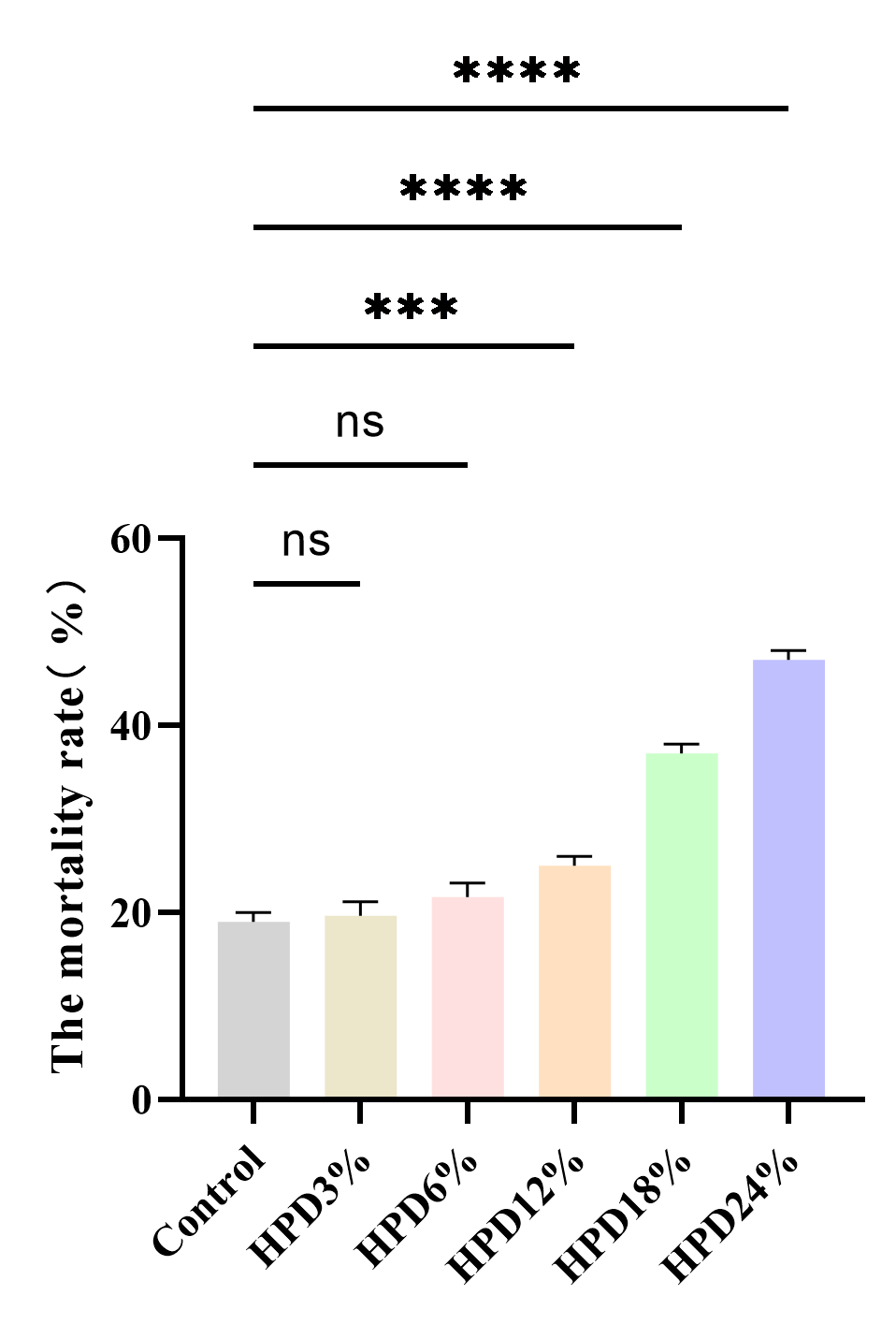

Supplement: Supplementary file 1 [file insects-16-00337-s001.zip › Fig S1.tif]
